# Deep undepleted human serum proteome profiling toward biomarker discovery for Alzheimer’s disease

**DOI:** 10.1186/s12014-019-9237-1

**Published:** 2019-04-17

**Authors:** Kaushik Kumar Dey, Hong Wang, Mingming Niu, Bing Bai, Xusheng Wang, Yuxin Li, Ji-Hoon Cho, Haiyan Tan, Ashutosh Mishra, Anthony A. High, Ping-Chung Chen, Zhiping Wu, Thomas G. Beach, Junmin Peng

**Affiliations:** 1 Departments of Structural Biology and Developmental Neurobiology, St. Jude Children’s Research Hospital, Memphis, TN 38105 USA; 2 Center for Proteomics and Metabolomics, St. Jude Children’s Research Hospital, Memphis, TN 38105 USA; 3 Banner Sun Health Research Institute, Sun City, AZ 85351 USA; 4 Present Address: Department of Laboratory Medicine, Nanjing Drum Tower Hospital, Nanjing University Medical School, Nanjing, 210008 Jiangsu China

**Keywords:** Alzheimer’s disease, Biomarker, Human blood, Plasma, Serum, Mass spectrometry, Proteomics, Proteome, Tandem mass tag

## Abstract

**Background:**

Blood-based protein measurement is a routine practice for detecting biomarkers in human disease. Comprehensive profiling of blood/plasma/serum proteome is a challenge due to an extremely large dynamic range, as exemplified by a small subset of highly abundant proteins. Antibody-based depletion of these abundant proteins alleviates the problem but introduces experimental variations. We aimed to establish a method for direct profiling of undepleted human serum and apply the method toward biomarker discovery for Alzheimer’s disease (AD), as AD is the most common form of dementia without available blood-based biomarkers in clinic.

**Methods:**

We present an ultra-deep analysis of undepleted human serum proteome by combining the latest 11-plex tandem-mass-tag (TMT) labeling, exhaustive two-dimensional liquid chromatography (LC/LC) fractionation (the 1st LC: 3 h for 180 fractions, and the 2nd LC: 3 h gradient per fraction), coupled with high resolution tandem mass spectrometry (MS/MS). AD (*n* = 6) and control (*n* = 5) sera were analyzed in this pilot study. In addition, we implemented a multiplexed targeted LC–MS3 method (TOMAHAQ) for the validation of selected target proteins.

**Results:**

The TMT–LC/LC–MS/MS platform is capable of analyzing 4826 protein components (4368 genes), covering at least 6 orders of magnitude in dynamic range, representing one of the deepest serum proteome analysis. We defined intra- and inter- group variability in the AD and control groups. Statistical analysis revealed differentially expressed proteins in AD (26 decreased and 4 increased). Notably, these altered proteins are enriched in the known pathways of mitochondria, fatty acid beta oxidation, and AGE/RAGE. Finally, we set up a TOMAHAQ method to confirm the decrease of PCK2 and AK2 in our AD samples.

**Conclusions:**

Our results show an ultra-deep serum discovery study by TMT–LC/LC–MS/MS, and a validation experiment by TOMAHAQ targeted LC–MS3. The MS-based discovery and validation methods are of general use for biomarker discovery from complex biofluids (e.g. serum proteome). This pilot study also identified deregulated proteins, in particular proteins associated with mitochondrial function in the AD serum samples. These proteins may serve as novel AD candidate biomarkers.

**Electronic supplementary material:**

The online version of this article (10.1186/s12014-019-9237-1) contains supplementary material, which is available to authorized users.

## Background

Cellular and biochemical components in blood play a central role in human physiology and their dynamic levels are considered to correlate with an individual’s healthy and diseased states [1, 2]. Blood is an exceptionally complex fluid, comprised of cells (i.e. red and white blood cells and platelets) and plasma (the liquid part) from which serum is collected after removing clotting factors with adequate coagulation. Human plasma/serum contains extraordinary diverse proteins, secreted from all types of cells and tissues for normal physiological function, leaked from damaged cells and tissues especially under disease conditions, or released from infectious organisms. Measuring various protein concentrations in plasma/serum is routine in clinical practice. The concentration dynamic range spans at least 10 orders of magnitude, from the most abundant albumin (~ 50 mg/ml) to cytokines of low abundance (e.g. 4.2, 7.4 and 11.2 pg/ml for interleukin-6, interleukin-17 and TNF-α, respectively) in normal individuals [3, 4]. This extremely high dynamic range raises a significant challenge for profiling the complete plasma/serum by a proteomics platform, commonly based on liquid chromatography-tandem mass spectrometry (LC–MS/MS). Depletion of highly abundant plasma proteins is often used to alleviate the dynamic range challenge, as the top 22 abundant proteins occupy approximately 99% of the total protein mass [1]. The depletion may be achieved by affinity columns immobilized with antibodies against the top abundant proteins [5–7]. However, there are multiple caveats associated with the depletion method: (i) the antibodies are never completely specific and may remove other nonspecific proteins; (ii) the depletion is performed under non-denaturing condition, leading to co-immunoprecipitation and removal of antigen-bound proteins; and (iii) the depletion step generates significant experimental variations [7].

Advances in mass spectrometry (MS)-based proteomics [8, 9], especially in LC separation power and MS resolution and scan rate, enable the profiling of more than 15,000 proteins (> 12,000 genes) from mammalian tissue samples [10, 11]. Protein quantification can be achieved through data dependent acquisition (e.g. label free method and stable isotope labeling) [12], as well as data-independent acquisition [13]. Tandem-mass-tag (TMT) is a commonly used stable isotope labeling method, which allows up to 11-plexed analysis [14, 15]. Although the accuracy of TMT measurement is often affected by ion co-elution-induced ratio compression, this issue is largely addressed by the MS3 method [16] or the combination of extensive LC fractionation, MS optimization, and computational correction [17]. With the success of tissue profiling [18, 19], we attempted to apply this latest TMT–LC/LC–MS/MS technology to analyze blood-based complex biofluids for Alzheimer’s disease (AD) biomarker discovery.

Following the discovery of putative biomarkers, it is necessary to validate these candidates in large clinical cohorts, usually by Ab-based approaches or targeted MS methods [2], such as selected single, multiple and parallel reaction monitoring (SRM, MRM, and PRM, respectively) [20, 21]. More recently, Triggered by Offset, Multiplexed, Accurate mass, high resolution, and Absolute Quantitation (TOMAHAQ) has been reported as an isobaric targeted method [22, 23]. For each targeted peptide quantification, TOMAHAQ implements a synthetic, TMT0-labeled peptide, which is used to trigger the quantification of native target peptide by MS3, based on a pre-selected offset mass. During the generation of MS3 spectra, synchronous precursor selection (SPS) can improve quantification accuracy by selecting pre-defined *b*- or *y* ions in MS2.

Alzheimer’s disease is the most common form of dementia and the sixth-leading cause of death in the US, affecting more than 5 million Americans with a healthcare cost of $236 billion in 2016 [24]. By 2050, AD patients are projected to reach 13.8 million in the US [24] and 100 million worldwide [25]. Currently, AD diagnosis is based on patient’s symptoms, memory and behavior tests, brain imaging, as well as post-mortem brain pathological assays [26, 27]. Blood-based biomarkers, however, are not available for AD, and most proposed candidates are derived from known disease mechanisms, such as Aβ and tau [28, 29]. Here we present the unbiased, large-scale profiling of human serum specimens, revealing consistent mitochondrial protein changes between control and AD samples.

## Methods

### Patient sample description

Human blood sera were collected from control (*n* = 5) and AD patients (*n* = 6), provided by the Brain and Body Donation Program at Banner Sun Health Research Institute, with approval for this study. Clinical and pathological diagnoses were based on established criteria [30]. All subjects consented to the study, and informed consent was obtained from each entrant. After clotting and centrifugation, the sera were frozen and stored at − 80 °C in aliquots of polyethylene tubes until use.

### Serum protein extraction and quantification

Human serum proteins were extracted in fresh lysis buffer [50 mM HEPES, pH 8.5, 8 M urea, and 0.5% sodium deoxycholate with 1 × phosphatase inhibitor cocktail (PhosSTOP, Sigma-Aldrich)]. The protein concentration was measured by the BCA assay (Thermo Fisher Scientific) and confirmed by Coomassie-stained short SDS gel as previously described [31]. The protein lysates were stored at − 80 °C in aliquots before use.

### Protein digestion and TMT labeling

The digestion and labeling were performed based on an optimized protocol [32, 33]. Quantified protein (~ 0.1 mg in the lysis buffer with 8 M urea) for each TMT channel was directly digested with Lys-C (Wako, 1:100 w/w) at 21 °C for 2 h, diluted four-fold to lower urea concentration to 2 M, and further digested with trypsin (Promega, 1:50 w/w) at 21 °C overnight. The digestion was terminated by the addition of 1% trifluoroacetic acid (TFA) with centrifugation. The supernatant was desalted with Sep-Pak C18 cartridge (Waters), and then dried by a speedvac vacuum concentrator. Each sample was re-dissolved in 50 mM HEPES, pH 8.5, reacted with TMT reagents, pooled equally, and desalted again before LC/LC–MS/MS.

### Extensive LC/LC-MS/MS analysis

The pooled TMT labeled peptides were resolved by offline basic pH reverse phase LC, and acidic pH reverse phase LC coupled with MS/MS analysis [34]. The setting of basic pH LC included a XBridge C18 column (3.5 μm particle size, 4.6 mm × 25 cm, Waters), buffer A (10 mM ammonium formate, pH 8.0), buffer B (95% acetonitrile, 10 mM ammonium formate, pH 8.0) [33], and a 3 h gradient of 15–35% buffer B. Each fraction was collected every minute, ending with a total of 180 fractions. In the acidic pH LC-MS/MS analysis, each previous fraction was analyzed on a column (75 µm × 25 cm, heated to 65 °C to reduce backpressure) coupled with a Q Exactive HF Orbitrap mass spectrometer (Thermo Fisher Scientific). Peptides were resolved by a 3 h gradient (buffer A: 0.2% formic acid, 5% DMSO; buffer B: buffer A plus 65% acetonitrile). MS settings included MS1 scans (60,000 resolution, 1 × 10^6^ AGC and 50 ms maximal ion time) and 20 data-dependent MS2 scans (410–1600 *m/z*, 60,000 resolution, 1 × 10^5^ AGC, ~ 150 ms maximal ion time, HCD, 32% normalized collision energy, and ~ 15 s dynamic exclusion).

### Identification and quantification of proteins by JUMP software suite

The bioinformatics processing of identification was carried out with our recently developed JUMP search engine, which combines the advantage of pattern- and tag-dependent scoring to improve sensitivity and specificity [35]. A composite target-decoy database was used to estimate false discovery rate (FDR) [36]. The protein database was generated by combining downloaded Swiss-Prot, TrEMBL, and UCSC databases and removing redundancy (human: 83,955 entries). Major parameters were precursor and product ion mass tolerance (± 15 ppm), full trypticity, two maximal missed cleavage, static mass shift for TMT tags (+ 229.16293 on Lys and N-termini) and carbamidomethyl modification (57.02146 on Cys), dynamic mass shift for oxidation (+ 15.99491 on Met), and three maximal modification sites. The resulting PSMs were filtered by mass accuracy, and then grouped by precursor ion charge state followed by the cutoffs of JUMP-based matching scores (J-score and ΔJn) to reduce FDR below 1% for proteins. When the same peptide is derived from numerous homologous proteins, the peptide was matched to the protein with the top PSM number, according to the rule of parsimony. The quantification was performed as previously described [17].

### Calculation of abundance index of identified proteins by PSMs

The absolute protein abundance index of serum proteome was calculated based on previously reported methods [37, 38], using the total number of PSMs matched to a particular protein, normalized by theoretically detectable peptides from the protein. It was derived by the formula: (the number of PSMs/the number of theoretically detectable peptides) × a scale factor. The scale factor was set to 5000, which generated abundance indexes that were roughly equivalent to protein copy numbers per cell during deep proteomics analyses.

### Evaluation of sample variations and principal component analysis

The measurement variation was analyzed according to intra- and inter-group replicates. The ratios of all proteins from the samples were modeled with a Gaussian distribution to evaluate standard deviation (SD). Principal component analysis (PCA) was used to visualize the differences among human disease groups. Relative expression of all proteins was used as input of PCA, using a R statistical analysis package (version 3.4.0) [39].

### Differential expression (DE) analysis, pathway enrichment and protein–protein interaction (PPI) analysis

DE analysis was determined by student *t* test in the following steps: (i) calculating *p* values and applying a threshold of 0.05; (ii) filtering by at least 1.5 fold of the standard deviation in the analysis; (iii) manually examining all proteins to remove proteins quantified by only one peptide.

Pathway enrichment analysis was used to infer functional groups of proteins enriched in a given pathway. The analysis was performed using Fisher’s exact test (*p* value) with the BH correction for multiple testing (BH FDR). Enriched pathways with FDR < 0.05 were considered statistically significant.

DE proteins were matched to a composite PPI database by integrating STRING (v10) [40], BioPlex [41], and InWeb_IM [42], including 18,515 proteins and 469,993 PPI connections. Modules in each protein cluster were defined as previously reported [18]. Modules were annotated by Gene Ontology, KEGG or Hallmark.

## TOMAHAQ targeted LC–MS3 analysis

The TOMAHAQ assay was based on the initially reported protocol [22]. Selected peptides were synthesized, purified (at least 95% purity), and dissolved in 20% acetonitrile. The peptides were labeled by a TMT0 reagent (Thermo Fisher Scientific), desalted, and spiked into the TMT11-labeled pooled samples. The amount of TMT0-labeled synthetic peptides was adjusted to ensure detection in MS1.

In the LC–MS3 analysis, the TMT0-TMT11 mixed samples were analyzed on a reverse phase LC coupled with MS3 analysis. The setting included a C18 column (50 µm × 15 cm, 1.9 μm particle size, heated to 65 °C to reduce backpressure), buffer A (0.2% formic acid, 5% DMSO) and buffer B (buffer A plus 65% acetonitrile) in a 1 h gradient of 10–35% buffer B at 250 nl/min, and an Orbitrap Fusion mass spectrometer (Thermo Fisher Scientific). The TOMAHAQ workflow comprises a sequence of decisions to prompt quantitative SPS-MS3 in multiple scans. In scan 1, survey MS1 scans (mass range: ± 50 *m/z* of target peptides, 60,000 resolution, 1 × 10^6^ AGC and 100 ms maximal ion time) were used to detect one TMT0 labeled, synthetic trigger peptide (± 15 ppm). If the intensity threshold (1 × 10^5^) was reached, the trigger peptide was fragmented in scan 2 (0.4 *m/z* isolation window, and ~ 35 NCE in CID) and detected by Orbitrap (15,000 resolution; 1 × 10^5^ AGC; 50 ms maximal ion time). A “Product Ion Trigger” function was used to compare the trigger peptide MS2 spectra to a pre-determined MS2 product ion list (± 10 ppm). If at least 6 product ions were matched, it trigged scans 3 and 4 to analyze the corresponding target peptide, using a pre-selected offset (peptide-specific, e.g. 5.01 *m/z* for *z* = 2 and two TMT tags). In scan 3, target MS2 was collected (0.4 *m/z* isolation window, ~ 35 NCE in CID, 15,000 resolution; 1 × 10^5^ AGC; 1000 ms maximal ion time). In scan 4, target MS3 was collected based on the previous MS2 and additional MS3 settings: Precursor Ion Exclusion (Low = 70, High = 5), Isobaric Tag Loss Exclusion (Reagent Tag Type = TMT to exclude “complement” MS2 ions), 0.4 *m/z* isolation window for 10 pre-defined MS2 product ions on a “Targeted Mass Inclusion List”, 55 NCE in HCD, 60,000 resolution, 1 × 10^5^ AGC, and 2,500 ms maximal ion time.

## Availability of data and materials

The mass spectrometry proteomics raw data have been deposited to the Proteome Xchange Consortium (http://www.proteomexchange.org) [43] via the PRIDE partner repository with the dataset identifier PXD011482.

## Results and discussion

### Multiplexed quantitative analysis of undepleted human serum proteome

**Fig. 1:**
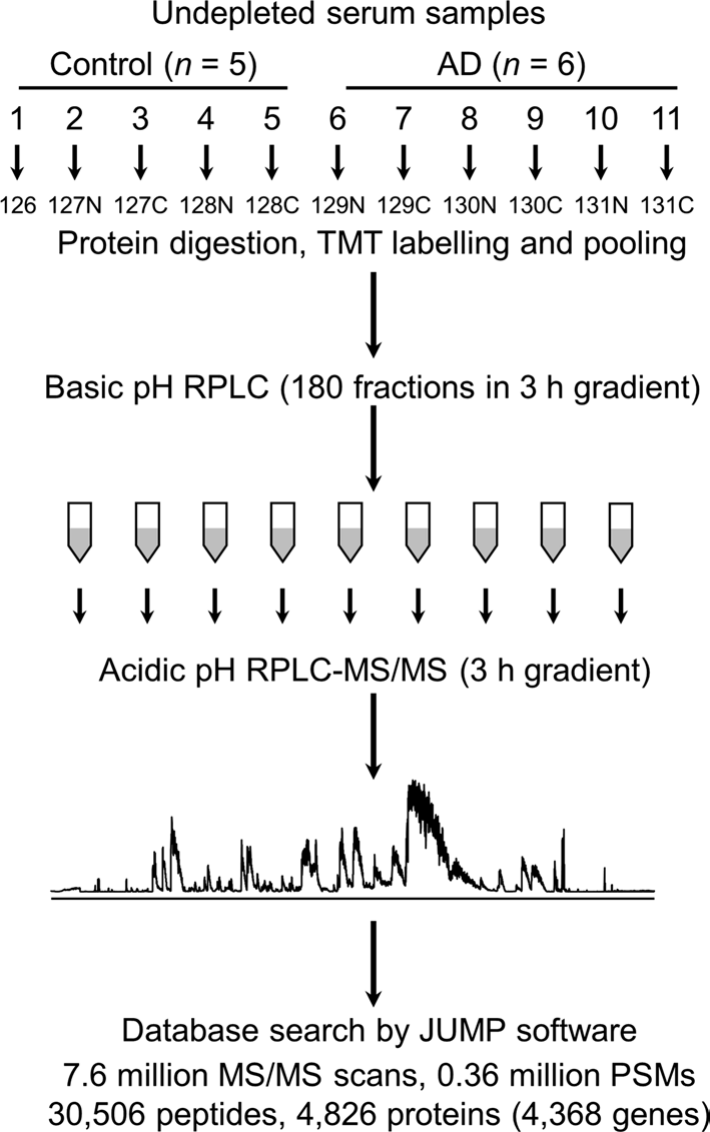
Experimental scheme of deep undepleted serum proteome analysis using TMT–LC/LC–MS/MS. AD and control serum samples were extracted, digested, labeled and pooled. The pooled peptide mixture was resolved in a 3 h gradient by basic pH RPLC, with fractions collected every minute (*n* = 180). Each collected fraction was subjected to the analysis by automated nanoscale acid pH RPLC, coupled with high resolution tandem mass spectrometry. All resulting data were analyzed by the JUMP software suite

A flowchart of the experiment is presented in Fig. 1, in which we profiled serum proteome without depletion by extensive TMT–LC/LC–MS/MS to maximize sensitivity and proteome coverage. Clinical characteristics of AD patients, together with gender- and age-matched control cases, are summarized in Additional file 1: Table S1. The whole serum protein extracts were denatured and trypsinized into peptides. The resulting peptides were differentially labelled with different TMT tags, and then equally pooled. The pooled peptides were separated by extensive offline basic pH reverse phase (RP) LC, collected into as many as 180 fractions to decrease the complexity in each fraction. We then used a long, high resolution column and 3 h gradient for acidic pH RPLC (totaling 540 h) coupled to the mass spectrometer. Taking account of maintenance time for LC, we spent approximately one month of instrument time to obtain ultra-deep analysis. In total, we collected 7.6 million MS/MS scans and accepted 0.36 million peptide-spectrum matches (PSMs) after database search, leading to the identification and quantification of 30,506 unique peptides, 4826 proteins with < 1% false discovery rate (FDR), corresponding to 4368 genes (Additional file 1: Table S2). The most abundant protein serum albumin (ALB) was identified by 47,006 PSMs (13.1% of all accepted PSMs), and the top 22 proteins occupied 262,008 PSMs (72.2% of the accepted PSMs). In spite of the presence of these exceedingly abundant species, we were still able to identify 3415 (70.8%) proteins with at least two matching peptides, and 3912 (81.1%) proteins with at least two PSMs. To our knowledge, this is one of most comprehensive quantitative analyses of human serum in a single experiment.

### Estimation of the minimal fraction number to achieve high serum proteome coverage

**Fig. 2:**
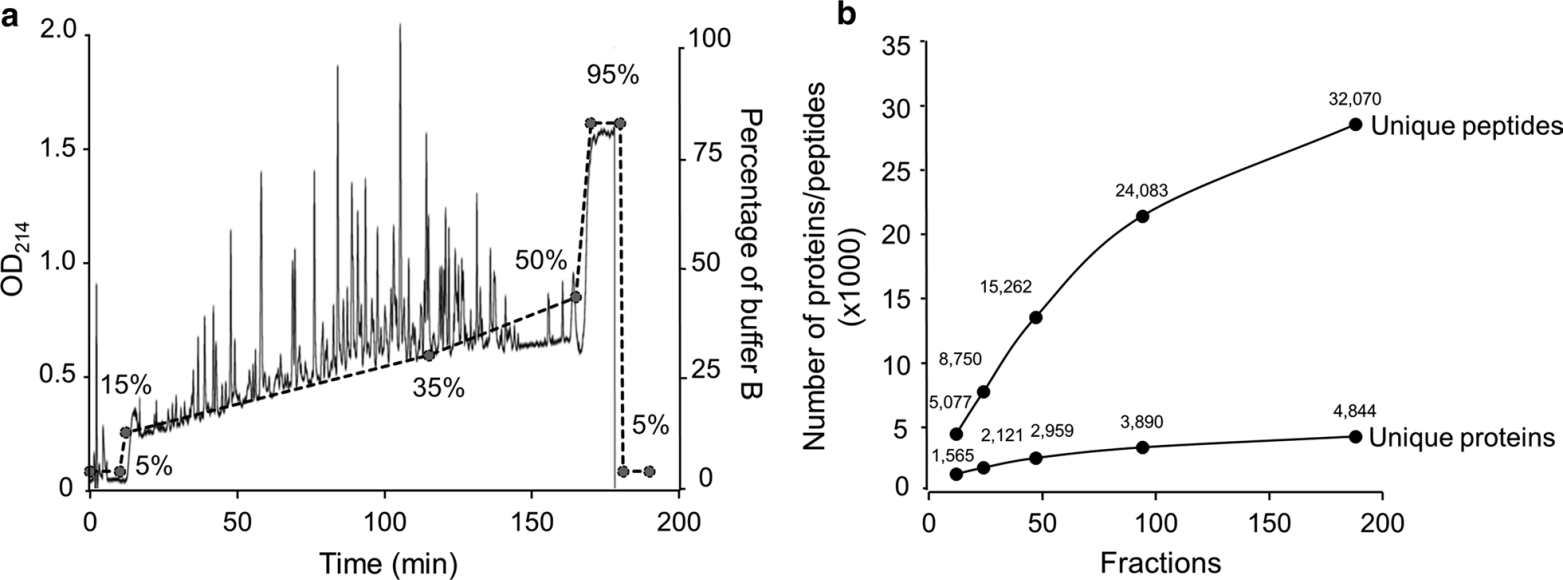
Basic pH RPLC elution profile and protein identification in individual fractions. **a** Extensive elution profile of pooled peptide mixture by basic pH RPLC. Peptides were eluted from a gradient buffer and monitored by UV absorption at 214 nm. Fractions were collected every minute. **b** Cumulative curve of total peptides and proteins by different combinations of fractions. For example, for the 90 alternative fractions, 24,083 peptides and 3890 proteins were identified

As the serum samples have different protein composition and dynamic range from tissue specimens, we attempted to evaluate the performance of the two dimensional LC and to optimize a strategy for serum proteome analysis. During the 3 h gradient of the 1st dimensional basic pH RPLC (15–35% buffer B, Fig. 2a), the majority of the peptides were eluted between 25 and 165 min. In this pilot analysis, we did not concatenate the 180 fractions because mixing fractions regenerates peptide complexity. Consistently, in the 2nd dimensional acid pH RPLC–MS/MS, most peptides/proteins were identified between fractions 25–165. However, the number of identified peptides were not evenly distributed, suggesting that concatenation may be a solution to equalize peptide content and improve analytical efficiency.

In shotgun proteomics, longer analytical time is generally rewarded with higher peptide/protein coverage until the saturation point is reached. Indeed, prior to the analysis of the half of fractions (*n* = 90, every alternative fraction), identified peptides increased with fractions in an approximately linear fashion (Fig. 2b). After 100 fractions, the slope appeared to decrease dramatically, implicating that the analysis was close to saturation. To enhance the throughput of this platform, it is possible to analyze ~ 4000 proteins with the half of these fractions to balance coverage and MS usage.

### Evaluation of sensitivity and dynamic range for the identified serum proteome

**Fig. 3:**
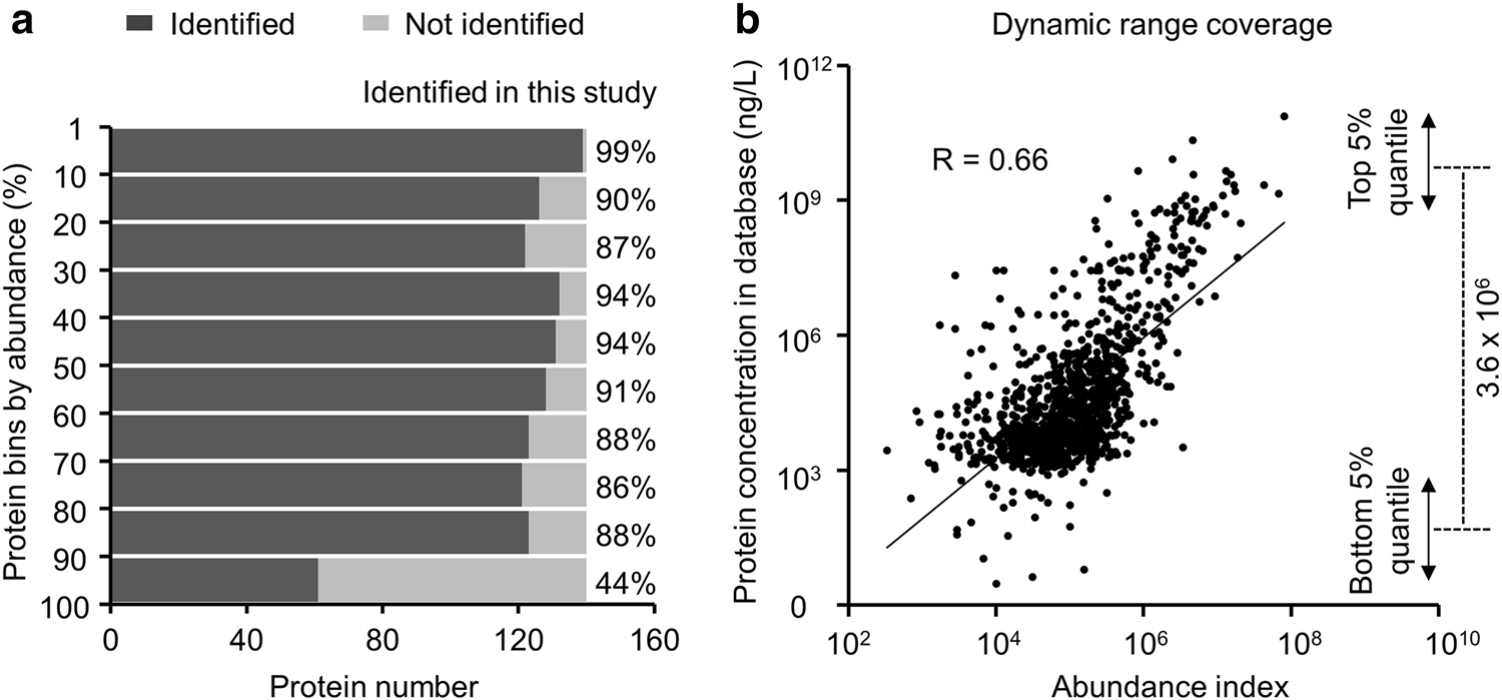
Estimation of the method sensitivity and dynamic range. **a** Comparison between our dataset and plasma proteome database. The plasma proteome database contains concentration information for a large set of proteins. We extracted these proteins with concentration and divided them into 10 equal bins. In each bin, protein percentage identified in our dataset are highlighted (e.g. 99% in the top bin). **b** Plot of known protein concentration in the plasma proteome database against absolute protein abundance index calculated in our dataset

To assess the sensitivity of our method, we compared our dataset with a public plasma proteome database, and focused on the 1399 proteins with reported plasma concentrations [44]. Out of these proteins in the database, we detected 1206 (86%) proteins in our analysis. While sorting the database proteins into 10 subsets by abundance (*n* = ~ 140 per subset) (Fig. 3**a**), our analysis identified at least 85% in the top 9 subsets, and the remaining < 15% proteins do not have sufficient tryptic peptides compatible with our method. Even in the 10th subset of proteins with the lowest abundance, we still detected 44% of these proteins. Together, this comparison indicates that only a very small portion of plasma proteins were below our detection limit, demonstrating high sensitivity of our TMT–LC/LC–MS/MS method.

We next computed the abundance index based on PSMs after size normalization (see “Methods”) and evaluated the dynamic range in our dataset. The abundance index is consistent with known protein concentrations in the plasma database (R = 0.66, Fig. 3b, Additional file 1: Table S3). As to the dynamic range we covered, serum albumin has the highest concentration (7.6 × 10^10^ ng/L), and cardiac type troponin T2 (TNNT2) has the lowest concentration (3.0 ng/L), spanning a range of more than 10 orders of magnitude. Conservatively, looking at the 5% top and bottom quantile, the estimated dynamic range is 3.6 × 10^6^ (Fig. 3b). The results indicate a broad dynamic range is covered by the deep analysis.

### Quality control analysis and intra- and inter-group variations in AD-control serum proteomes

**Fig. 4:**
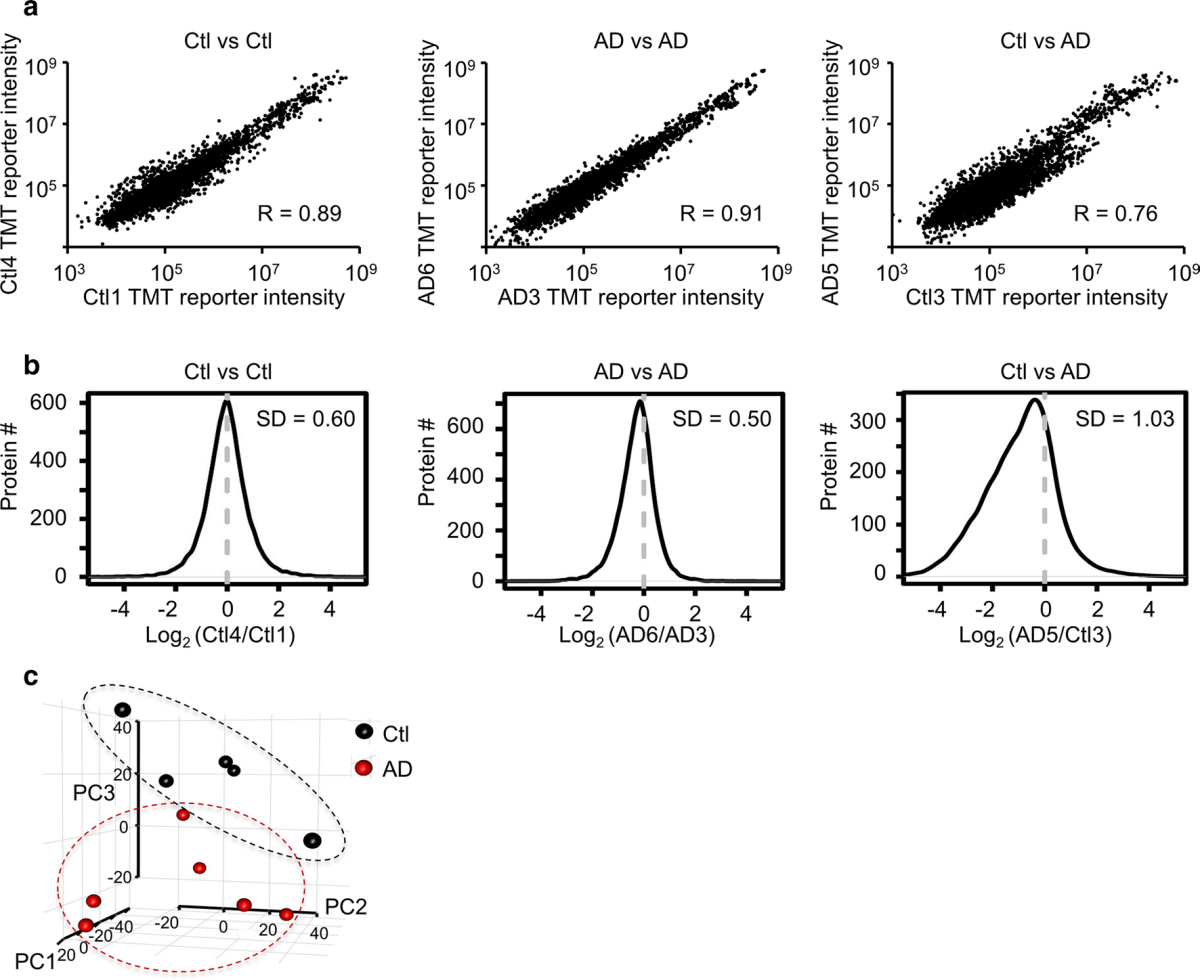
Statistical analysis to determine quality and intra/inter-group variations of serum proteome. **a** Representative comparisons of intra- and inter-group variations based on TMT reporter intensities for identified proteins in AD and control cases. **b** Histograms based on protein log_2_ ratios, fitted to normal distribution to derive standard deviation. **c** Principal component analysis of identified proteins

We performed quality control analysis by comparing any of the two samples in the 11 quantified cases, and evaluated intra- and inter-group variations in AD and control cases. To compare the samples, we plotted the TMT reporter intensities for all identified proteins (Fig. 4a). All two-sample comparisons showed a consistent and reproducible pattern (R of at least 0.7). For example, R values of the Ctl4/Ctl1, AD6/AD3, and AD5/Ctl3 were 0.89, 0.91 and 0.76. For the above comparisons, we also derived the log_2_ratio values for all proteins to generate the histograms, which were largely fit into normal distribution to generate standard deviation (Fig. 4b). As anticipated, the intra-group (e.g. Ctl4/Ctl1 and AD6/AD3) and inter-group standard deviation (e.g. AD5/Ctl3) were 0.60, 0.50 and 1.03, respectively, consistently with the R values (Fig. 4a).

To fully compare intra- and inter-group variations, we obtained standard deviation values for all two-sample comparisons (*n* = 10 for the control group, *n* = 15 for the AD group, and *n* = 30 for the AD/control group). The averages of standard deviations in the control, AD, and AD/control comparisons were 0.75 ± 0.15, 0.73 ± 0.12, and 0.78 ± 0.14, respectively. Although the inter-group had slightly larger variations than the intra-group comparisons, there is no statistically significant difference, which may be due to the small cohort size, or large confounding factors, such as gender, age, genetic background, clinical treatment, and other pre- and post-sample collection variance [29, 45]. However, three-dimensional principal-component analysis (PCA) of all quantified proteins displayed the separation of control and AD cases (Fig. 4c), confirming the reproducibility of the analysis.

### Serum proteomics reveals deregulation of mitochondrial pathways in AD cases

**Fig. 5:**
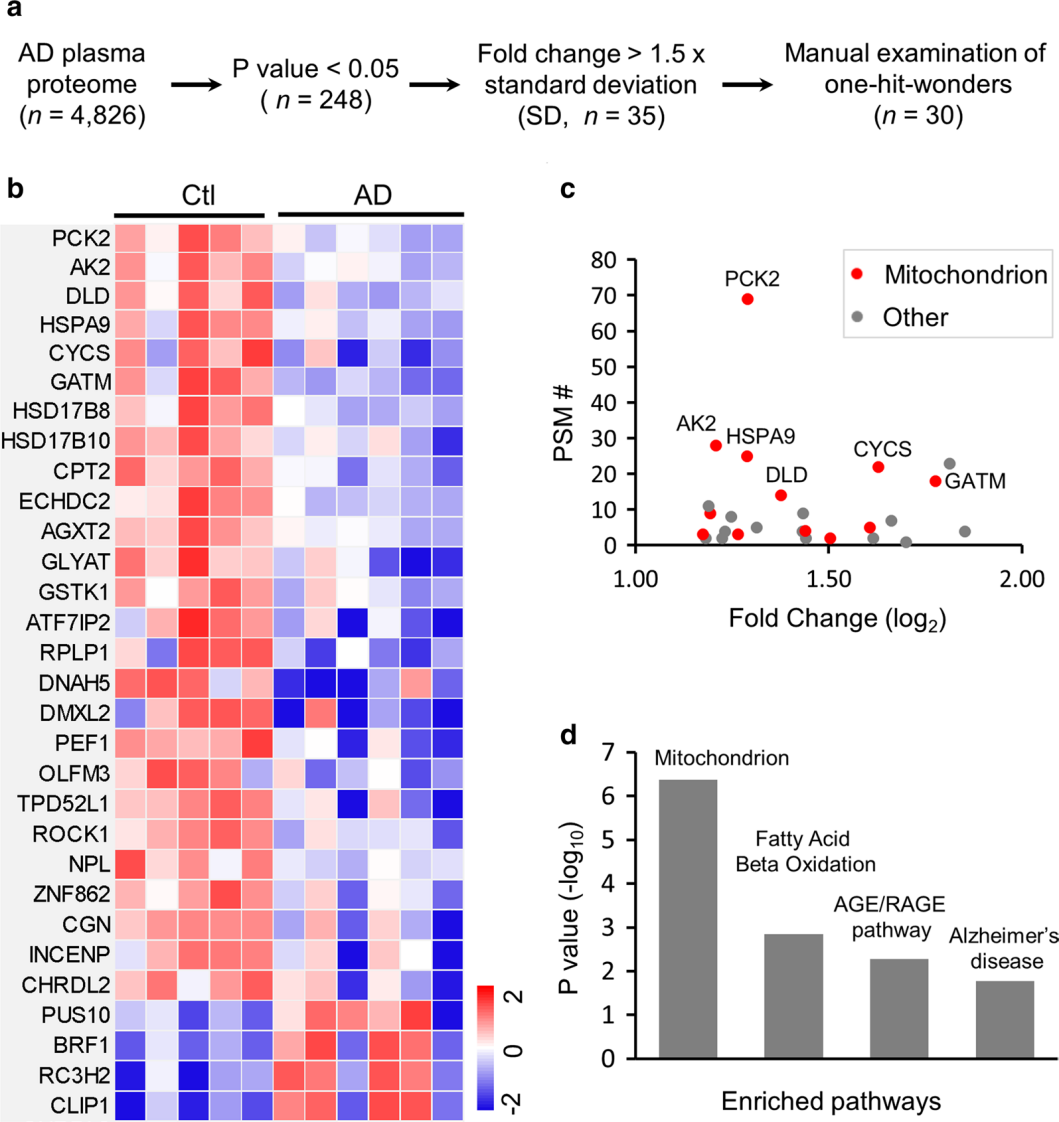
Analysis of whole serum proteome reveals mitochondrial associated signaling pathway. **a** Summary of the computational pipeline for serum proteome analysis. **b** Heatmap of differentially expressed proteins from control and AD samples (*p* < 0.05). **c** Fold change and PSM-based abundance of downregulated proteins in AD. **d** Functional annotations of enriched differentially expressed serum proteome

To study serum proteome alterations in AD, we established a computational pipeline by integrating differentially expressed (DE) analysis with pathway enrichment [11, 18] (Fig. 5a). Out of 4826 proteins identified in the serum samples, we initially identified 248 DE proteins (*p* < 0.05), which were filtered by log_2_(AD/Ctl) changes (1.5 fold of average standard deviation at 0.75, equal to 1.125 on the log2 scale, equivalent to 2.2 fold change), resulting in 35 DE proteins. After manual examination to remove one-hit-wonders, we accepted a final list of 30 proteins (Additional file 1: Table S3), shown in a heat map representing log_2_ratios between AD and control samples (Fig. 5b). Whereas 4 (13%) proteins (PUS10, BRF1, RC3H2, and CLIP1) showed higher expression in the AD than the control, 26 (87%) proteins had lower expression in AD. Strikingly, out of these downregulated proteins, 12 proteins are localized in mitochondria, including some abundant proteins (e.g. PCK2, AK2, HSPA9, CYCS, DLD, and GATM, with at least 14 PSMs) (Fig. 5c).

Consistently, the 30 DE proteins were enriched in mitochondria-related pathway, as well as the signaling of fatty acid beta oxidation and AGE/RAGE (Fig. 5d). Interestingly, several proteins (HSPA9, CYCS, DLD, and GATM) were also enriched in various pathways related to Alzheimer’s disease [46–49]. Finally, we superimposed the DE proteins onto PPI network to extract functional modules that are assembled by interacting proteins to form functional units at a systems level. The PPI network was curated from the most commonly used databases, STRING [40], BioPlex [41], and InWeb_IM [42]. Computational analysis identified 3 PPI modules, all related to mitochondrial function, including mitochondrial envelope (CYCS and GSTK1), intermembrane space (GATM and AGXT2), and matrix (AK2, DLD, HSPA9, HSD17B10, HSD17B8, and ECHDC2). Mitochondrial failure has been long proposed to play an important role in the development of Alzheimer’s disease [50, 51]. The master mitochondrial regulator PGC-1α [52] was reported to be dysregulated in AD brain during the progression of neuropathology and dementia, leading to the downregulation of mitochondrial genes including PCK2 [53], supporting our proteomic findings. Thus, comprehensive profiling of serum proteome revealed the change of key mitochondrial proteins in AD that may be relevant to disease development.

In this deep proteomics analysis, we also detected tau and APP proteins in the samples. However, these proteins did not show statistically significant difference between the control and AD samples, partially due to the limited sample size. Recently, Nakamura et al. developed an approach to measure plasma Aβ by immunoprecipitation (IP) and MS, and proposed an AD composite biomarker based on (APP)_669–711_/Aβ_1–42_ and Aβ_1–40_/Aβ_1–42_ ratios [28]. The composite biomarker displayed high performance for predicting brain Aβ burden, and high correlation with Aβ_1–42_ in cerebrospinal fluid. Without the IP enrichment, the detailed ratio analysis could not be performed in our dataset. The IP-MS approach may be used to improve sensitivity for targeted biomarker candidates.

### TOMAHAQ-based multiplexed approach for target validation in AD samples

**Fig. 6:**
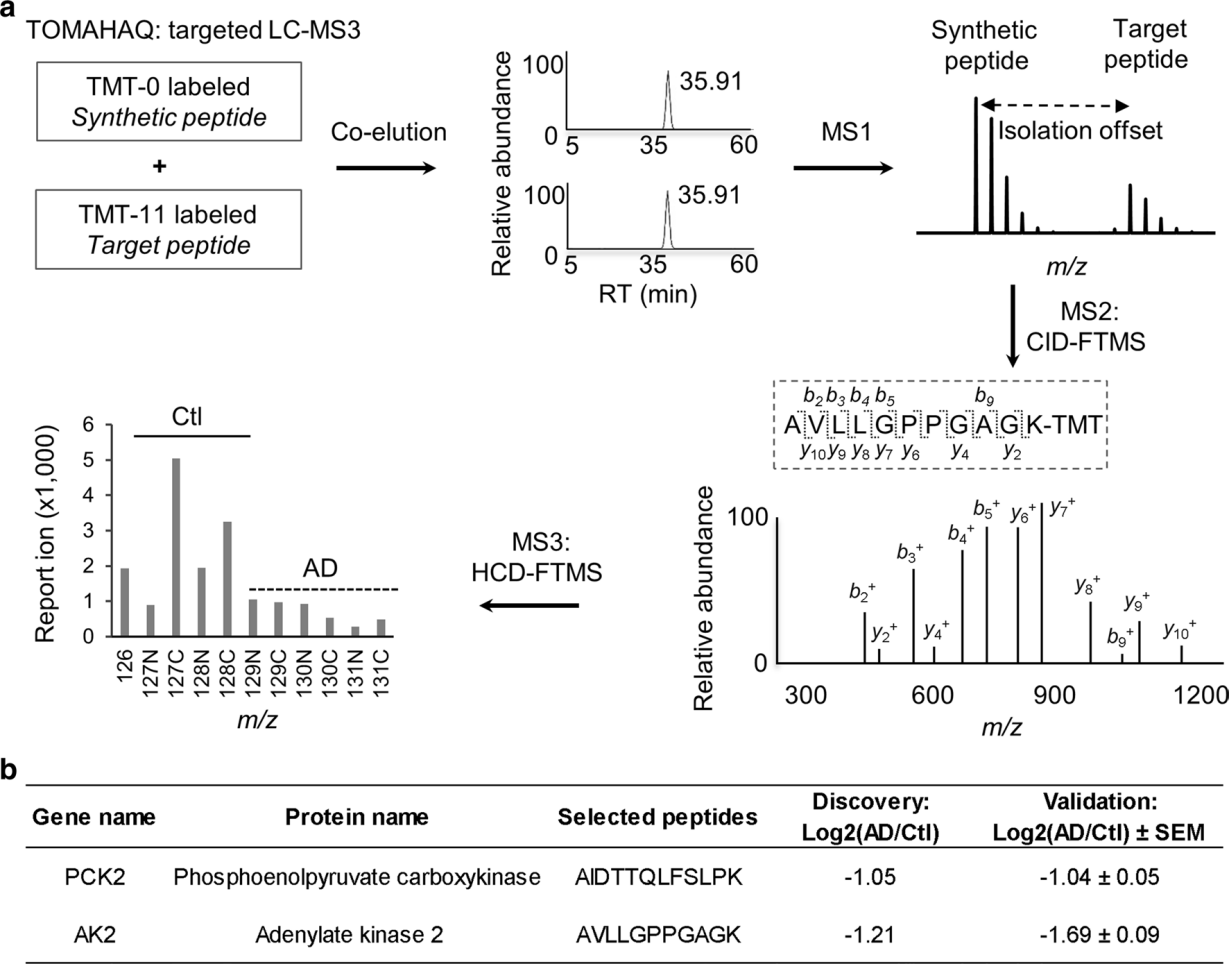
Validation of selected proteins by TOMAHAQ targeted LC-MS3 method **a** TOMAHAQ used synthetic trigger peptides which were spiked into a mixture of multiplexed samples. Monitoring trigger peptides enabled quantification of target peptides. MS3 analyses of the target peptides were based on pre-defined *b* or *y* ions from target MS2 spectra, and the resulting reporter ions were used for quantification. **b** Validation of known candidate proteins PCK2 and AK2

Finally, we utilized a TOMAHAQ-based LC–MS3 assay to validate two mitochondrial proteins AK2 and PCK2 which differentially expressed in our LC/LC–MS/MS discovery study. Both candidates were found to be down regulated in AD, with Log_2_ (AD/control) values of − 1.05 and − 1.21. In this validation assay, we synthesized two peptides as internal standards. The synthetic peptides were labeled with the TMT0 reagent, and then mixed with endogenous samples labeled with 11-plexed TMT reagents. TOMAHAQ allows simultaneous and accurate quantification of peptides across 11 samples in one assay. During the LC–MS runs, TMT0-peptides were always detected to trigger the measurement of their corresponding native peptides by MS3 spectra. The reporter ions of native peptides in the MS3 spectra were used for accurate quantification (Fig. 6a, see details in “Methods”). Consistently, the levels of PCK2 and AK2 in the AD cases were significantly lower, with Log_2_ (AD/control) values of − 1.04 ± 0.05 and − 1.69 ± 0.09, respectively (Fig. 6b), when compared to the control cases. This multiplexed method may be used for sensitive and accurate quantification of selected targets in large-scale clinical validation in the future.

## Conclusions

We identified 4826 proteins and demonstrated high proteome coverage, sensitivity and reproducibility, as well as multiplexed targeted assays. Although extensive fractionation and long instrumentation time were employed in this pilot study, we propose to achieve similar results of ~ 4000 proteins within a reasonable time frame. This extensive TMT–LC/LC–MS/MS platform will be of general application for the measurement of complex clinical specimens. Remarkably, even in this small cohort, we identified consistent changes of 30 proteins in AD specimens compared to the non-dementia controls, in which 12 proteins were clustered to the mitochondria-related pathway. These novel protein signatures may be related to AD progression and have potential to be followed as biomarkers in a large scale investigation, possibly by the TOMAHAQ-based LC–MS3 assay.

To our knowledge, this study (30,506 peptides from 4826 proteins) represents one of the deepest, undepleted serum proteome profiling experiments from human biofluid. Previous studies usually attempted to increase the serum/plasma proteome coverage by immunodepletion of abundance proteins and extensive separation [4]. In 2006, the combination of immunodepletion, chemical fractionation (isolating cysteinyl- peptides and glycol-peptides) and LC/LC–MS/MS, allowed the identification of 22,267 peptides from 3654 different proteins. In 2011, human plasma proteome datasets were compiled to produce a non-redundant list of 1929 proteins (20,433 peptides) of high confidence [54]. In 2015, with the advance of better fractions and instrumentation, about 4600 proteins were analyzed in human plasma by immunodepletion, isobaric labeling and LC/LC–MS/MS. In 2017, the human plasma proteome draft included 3509 proteins identified at least two peptides, and about 1300 additional ambiguous proteins [55]. The drawbacks of immunodepletion are the removal of non-targeted proteins, associated quantitative variability, and the cost of the antibody cartridge [7]. Our study demonstrates the possibility to achieve deep analysis without the step of immunodepletion. However, all of these deep plasma/serum profiling experiments were time consuming due to a large number of fractions, which are not well suited for large clinical studies. Alternatively, a single-run, label-free protocol was introduced for rapid analysis of hundreds of plasma proteomes, and with additional pre-fractionation, interpretation of 1000 proteins became possible [56]. Other approaches, such as SWATH, was used to quantify more than 300 plasma proteins in 232 plasma samples [57]. Furthermore, the throughput of profiling of biofluids can be increased by sample multiplexing, such as iTRAQ/TMT labeling [58]. Here, we adapted the TMT-derived TOMAHAQ method for targeted protein analysis. The integration of deep proteome coverage by extensive TMT–LC/LC-MS/MS in the discovery phase, and targeted measurement by TOMAHAQ in the validation phase, will represent a balance between comprehensive profiling and analytical time.

## Additional file

**Additional file 1: Table S1**. Summary of human cases used in this study. **Table S2**. Serum proteome profiling of AD and control cases by TMT–LC/LC-MS/MS. **Table S3**. Dynamic range analysis based on plasma proteome database concentration and MS-derived abundance index. **Table S4**. Differentially expressed serum proteins in AD and control cases by TMT–LC/LC-MS/MS.

## Abbreviations

AD: Alzheimer’s disease
MS: mass spectrometry
TMT: tandem mass tags
LC/LC–MS/MS: two dimensional liquid chromatography coupled with tandem mass spectrometry
TOMAHAQ: triggered by offset multiplexed accurate mass high resolution absolute quantification
